# How to Exercise

**Published:** 1882-05

**Authors:** 


### HALL'S JOURNAL OF HEALTH AND MISCELLANY.

MONTHLY.

E. H. GIBBS, A. M., M. D., EDITOR.

FOUNDED IN 1854.

### HOW TO EXERCISE.

PROPER and appropriate exercise is essential to health. There can be no healthy development without it. No subject is so much talked about and so little understood by most persons. Every one can discourse glibly on the subject, and in a general way prescribe it for every ailment. Twenty years ago, the hobby was cold-water bathing; ten years later, warm water was substituted for cold; then the gymnasium, with its dumb-bells and "lifting" machines—"health lifts," as they are called; and I think very properly, because no device, save dumb-bells, could lift a man's health and carry it away more effectually.

Strictly speaking, cold bathing may not be considered exercise; but I desire to say a few words on that subject. There are many mothers even now who bathe their infants in cold water every day, in the belief that it is a sanitary practice. It seems to me that a little reflection will cause any intelligent mother to abandon it at once. The infant is a delicate and sensitive creature, who has but three chances in four of ever getting out of his swaddling clothes under ordinary treatment. Were he intelligently cared for, his chances of reaching manhood would probably be ninety-nine in a hundred. But the average baby has his feet forced into shoes several sizes too small for him; his dress "commences low and ends high," leaving shoulders and knees exposed to cold air; he has less than half enough food to eat, and hardly a thimbleful of pure cold water to drink during the day; if he survives these tortures, a few cold-water baths often suffice to chill the blood so that it ceases to flow; and, finally, the responsibility for this cruel ignorance is sacriligiously attributed to Providence.

The gymnasium would be a valuable institution if all who go there to exercise would limit their gyrations and contortions within reasonable bounds. But there is always more or less rivalry in the performance of difficult feats, and the actors are often goaded on beyond the point of safety. Hundreds of young men have been broken down in this way, beyond the hope of restoration to health. All bodily motion is induced by contraction or extension of the various muscles of the body at command of the will; now if the individual becomes excited and eager to excel in some difficult feat, he forgets himself; that is, reason for the time loses control of him, and he over-exerts himself. The muscles have been so long on the stretch that they are tired out and lose their elasticity and have not the power to regain it. A man who has had much of this sort of experience may display some muscles of abnormal development, but it is often found that the material has been taken from points where it was needed far more.

It is perhaps better to take no exercise than to carry the thing to excess; but it should be an easy matter to regulate the amount to the requirements of the individual. If this is done its effects are marvelous. A few simple rules are sufficient. Never exercise to the extent of great fatigue. Stop short of that, and you are always gaining strength; go beyond, and you lose strength in proportion. The best exercise is horseback riding, because it keeps all the muscles in motion, *involuntarily*; the horse lends you his muscles. Walking is the next best method; all the muscles are brought into use, but it requires an effort of the will to maintain the motion, and this detracts from its value. Every one has noticed how much more a sprained wrist, for example, is relieved when rubbed briskly by another person than when rubbed by the other hand of the patient. There is something soothing in passivity. It is said that lazy people live the longest, and that may be the reason.

### NOTICE.

The office of Hall's Journal of Health will from May 1st be at 135 Eighth street, near Broadway. This change of location is rendered necessary by our increasing business, requiring more room than ever before since the Journal of Health was established in 1854. The issue of June will be larger than any that has ever preceded it, and will contain articles of great interest to the people. Among other things, we expect to have ready for publication a novel plan for preventing floods along the Mississippi River. It is expected that this plan will meet the approval of an eminent civil engineer of New York, who is now investigating it. Commencing with the June number, we shall publish also a series of interesting articles on the "Relation of Foods to Health."

